# Blood and adipose tissue DNA methylation in adults born preterm with a very low birth weight – a sibling comparison study

**DOI:** 10.1080/17501911.2025.2583893

**Published:** 2025-11-17

**Authors:** Helena H Hauta-alus, Justiina Ronkainen, Juho Kuula, Darina Czamara, Anni Heiskala, Samuel Sandboge, Johan Björkqvist, Nina Kaseva, Katri Räikkönen, Kirsi H Pietiläinen, Sylvain Sebert, Eero Kajantie

**Affiliations:** a Clinical Medicine Research Unit, MRC Oulu, Oulu University Hospital and University of Oulu, Oulu, Finland; b Public Health Research Team, Population Health Unit, National Institute for Health and Welfare (THL), Helsinki, Finland; c Research Program for Clinical and Molecular Metabolism (CAMM), Faculty of Medicine, University of Helsinki, Helsinki, Finland; d Research Unit of Population Health, University of Oulu, Oulu, Finland; e HUS Medical Imaging Center, Department of Radiology, University of Helsinki and Helsinki University Hospital, Helsinki, Finland; f Department of Genes and Environment, Max-Planck-Institute of Psychiatry, Munich, Germany; g Psychology/Welfare Sciences, Faculty of Social Sciences, University of Tampere, Tampere, Finland; h Department of Psychology, University of Helsinki, Helsinki, Finland; i Department of Obstetrics and Gynecology, Helsinki University Hospital, Helsinki, Finland; j Obesity Research Unit, Research Program for Clinical and Molecular Metabolism, Faculty of Medicine, University of Helsinki, Helsinki, Finland; k Healthy Weight Hub, Abdominal Center, Helsinki University Hospital and University of Helsinki, Helsinki, Finland; l Department of Clinical and Molecular Medicine, Norwegian University of Science and Technology, Trondheim, Norway; m Children’s Hospital, Pediatric Research Center, University of Helsinki and Helsinki University Hospital, Helsinki, Finland

**Keywords:** Epigenome-wide association studies (EWAS), DNA methylation, adipose tissue, preterm birth, very low birth weight (VLBW)

## Abstract

**Background:**

Preterm birth and very low birth weight (VLBW; <1500 g) increase risks for poor health outcomes, potentially mediated by epigenetic modifications such as DNA methylation (DNAm). We hypothesized that DNAm differs between VLBW adults and their siblings in blood and adipose tissue.

**Methods:**

We studied 75 adults born preterm with VLBW and 73 same-sex sibling controls from the Adults Born Preterm Sibling Study. DNAm at cytosine–guanine dinucleotide (CpG) sites in blood and adipose tissue was assessed using Illumina EPIC 850K at a mean age of 29 years. Biological pathways were investigated with QIAGEN ingenuity pathway analysis (IPA).

**Results:**

No differences were observed in blood DNAm. In adipose tissue, 458 CpG sites were differentially methylated (FDR *p* < 0.05) between VLBW and siblings. Top sites were annotated to genes related to lipid metabolism (cg00264176 (*FADS2)*, 0.077 (0.007), FDR *p* = 3.24 × 10^−14^) and neural development (cg08277679 (*KIF26A)*, 0.053 (0.005), FDR *p* = 8.22 × 10^−12^). IPA identified enrichment for 81 pathways (FDR *p* < 0.05).

**Conclusion:**

Our results suggest tissue-specific DNAm differences in VLBW adults compared to their siblings. The changes cluster in pathways related to lipid metabolism, neurodevelopment, and cardiometabolic regulation, suggesting lasting tissue-specific epigenetic modifications in VLBW adults.

## Plain Language Summary

This study looked at how being born very early and very small (birth weight less than 1500 g) might affect long-term health through changes in genetic material DNA. We focused on a process called DNA methylation (DNAm). DNAm affects how actively genes work. We compared 75 adults born very small and premature to 73 of their siblings.

We analyzed DNAm in blood and fat tissue when participants were 29 years old. No differences were found in blood samples. However, in fat tissue, we found 458 spots on the genetic material DNA that were activated differently in VLBW adults compared to their siblings. These changes were linked to genes involved in fat processing, heart health, and brain development.

The findings suggest that being born early and very small cause lasting changes in how genes work in fat tissue. This could explain why adults who are born very small have higher risks for heart and metabolic diseases. These insights may help guide future treatments to improve health outcomes for people born premature or small.

## 1. Introduction

Preterm birth (<37 gestational weeks) and low birth weight (<2500 g) have been associated with increased cardiometabolic risk factors, such as impaired glucose regulation, high blood pressure [1–3], and manifest disease including type 2 diabetes and cardiovascular diseases [4–7]. The Developmental Origins of Health and Disease (DOHaD) proposes [8,9] that early life exposures cause permanent alterations in the structure and function of organs and, consequently, affect cardiometabolic health. Although such associations are widely replicated, the underlying mechanisms remain unclear [10].

One suggested mechanism between early life events and later health is epigenetic changes [11,12]. Although epigenetic changes may be modified by early life exposures, they can remain relatively stable across life. The most studied epigenetic modification is DNA methylation (DNAm). In large meta-analyses, several differentially methylated sites have been identified to be associated with gestational age [13] and birth weight [14]. Some of these associations last into later life, although few studies have assessed DNAm in adults according to gestational age at birth or birth weight [15,16]. Few studies have investigated tissue-specific epigenetic modifications [17–19], as most have relied on blood samples, especially cord blood [13,14]. Small-scale studies indicate differential methylation in placental tissue of preterm births [20,21], and small for gestational age (SGA) births [22]. Epigenetic research in target tissues is scarce, and whether these birth conditions cause long-term alterations in adipose tissue DNAm is unknown. However, DNAm in fat tissue seems to associate with adiposity and blood lipid levels [23,24], with linkages to gene expression, glucose metabolism [25,26] and type 2 diabetes [27], which as mentioned, are also associated with the long-term morbidity of premature birth.

Genotype and the living environment of study participants, such as family’s sociodemographic status [28,29], may confound DNAm studies. To address this, we examined epigenome-wide DNAm of 75 adults born preterm with a very low birth weight (VLBW; <1500 g) and their 73 same-sex sibling controls in the Adults Born Preterm Sibling study (Sibling) [30]. DNAm was assessed in blood and adipose tissue using the Illumina Infinum Methylation EPIC array, which measures more than 850 000 methylation cytosine–phosphate–guanine (CpG) sites across the genome. In the Sibling study, we previously observed higher blood glucose and free fatty acid concentrations and increased odds for impaired glucose regulation in adults at VLBW compared with their sibling controls [31], as well as differences in body composition [32] and adipose tissue quality via magnetic resonance imaging (MRI) [33]. In the current study, our aim was to examine whether DNAm in blood and abdominal adipose tissue differs between adults born at VLBW compared with their sibling controls by using an epigenome-wide association study (EWAS) approach. Further, we sought to identify possible underlying biological mechanisms between early childhood exposure of VLBW, DNAm, and later health outcomes by Ingenuity pathway analysis.

## 2. Materials and methods

### 2.1. Participants

**Figure 1.**
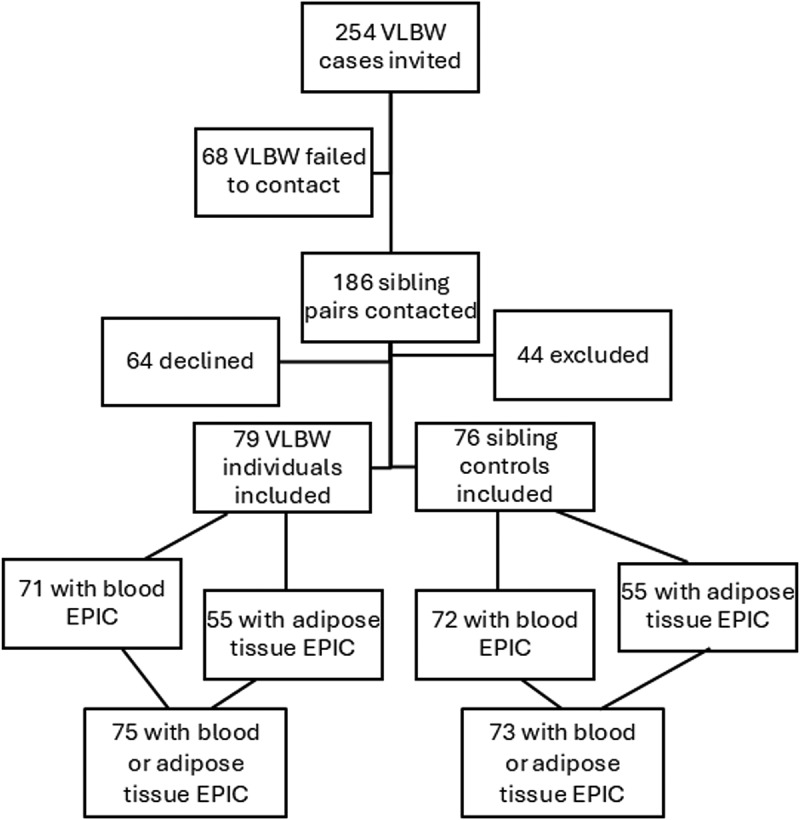
Flow chart of the study participants. VLBW, very low birth weight; EPIC, Infinium Human Methylation EPIC BeadChip array (850 K).

Detailed description of the Sibling study has been provided earlier [30,33,34]. The Sibling study comprises data from 79 adults born preterm at VLBW and 76 same-sex siblings serving as controls, all born between years 1976 and 1996 (34) (Figure 1). Most of the individuals with VLBW (*n* = 51) were identified through the Finnish Medical Birth Register of all births in Hospital Districts of Uusimaa, Varsinais-Suomi and Pirkanmaa in 1987–1990. Additional 22 individuals were recruited from the Helsinki Study of Very Low Birth Weight Adults (HeSVA) [2] and 6 individuals from the Preterm Birth, Pregnancy and Offspring Health in Adult Life (ESTER) study [35]. The HeSVA and ESTER studies comprise individuals born between 1978 and 1985 at the Children’s Hospital in Helsinki and between 1985 and 1989 in Northern Finland (provinces of Oulu and Lappi), respectively.

We excluded study participants who had an endocrine disorder or sensorineural impairment associated with preterm birth [30]. Two pregnant participants were offered permission to participate after pregnancy, but they declined. VLBW participants were born before the gestational week 37 with VLBW defined as birth weight <1500 g. Inclusion criteria for sibling–control were same sex, a maximum age difference of 10 years, and born at term. However, retrospectively from medical birth records, we discovered that four sibling controls were born preterm (at gestational weeks 35.9–36.9) including two late preterm (34–36 gestational weeks) and two early term (37–38 gestational weeks) participants with normal birth weight, and two sibling controls born at term but SGA (birth weight < −2.0 SDS [standard deviation score, based on national growth curves] [36]) (for both cases, birth weight was above −2.68 SDS). These individuals were included in the analyses, but additional sensitivity analyses were conducted excluding the controls born preterm or SGA.

DNA from blood samples was available for 146 individuals, and after DNAm quality control (QC), 3 were excluded, resulting in 71 adults at VLBW and 72 sibling controls. DNA from adipose tissue was available from 113 individuals, and after QC, 3 were excluded, resulting in 55 adults at VLBW and 55 sibling controls. Altogether 75 adults at VLBW with their 73 siblings had DNAm data available from blood or adipose tissue.

The study was conducted in accordance with the guidelines of The Declaration of Helsinki and approved by The Coordinating Ethics Committee of the Hospital District of Helsinki and Uusimaa (168/13/03/00/2013). All participants signed informed consent. The tissue biopsies were discussed separately with the possibility to withdraw from that part of the study. Metadata of our study can be found in Qvain repository following FAIR principles (https://doi.org/10.23729/fd-8e6178a7-f368-3d38-a28b-e16c911a6408).

### 2.2. Clinical measurements and tissue samples

Individuals born with VLBW and their sibling controls underwent extensive clinical measurements between 2014 and 2017 at a mean age of 29 years ranging from 19.5 to 38.7 (4.8 IQR) years. In addition to comprehensive clinical measurements, we collected blood samples as well as adipose and muscle tissue biopsies. A needle aspiration biopsy was obtained paraumbilically from superficial subcutaneous abdominal adipose tissue under local anesthesia. Samples were macroscopically inspected, divided into smaller aliquots and snap-frozen in liquid nitrogen.

To perform a 2-h 75 g oral glucose tolerance test (OGTT), peripheral venous blood samples were collected at baseline (after overnight fasting), 30, 60, and 120 min, after ingestion of a solution containing 75 g of glucose, for analyzing glucose and insulin concentrations. We applied the World Health Organization (WHO) criteria for diagnosing impaired glucose regulation: 1) Type 2 diabetes was defined as a fasting plasma glucose level of ≥7.0 mmol/l or a 2-h plasma glucose level of ≥11.1 mmol/l; 2) Impaired glucose tolerance was identified when fasting plasma glucose was <7.0 mmol/l and the 2-h value ranged between 7.8 and 11.0 mmol/l; 3) Impaired fasting glucose was defined by a fasting plasma glucose level between 6.1 and 6.9 mmol/l, with a 2-h plasma glucose level remaining below 7.8 mmol/l [31].

### 2.3. Dna methylation

DNA was extracted from whole blood samples and adipose tissue at the University of Helsinki by Allprep Universal DNA/RNA/miRNa kit according to the manufacturer’s protocol (Qiagen, Germany). DNA concentration was measured by Nanodrop and Qubit. DNAm was determined with the Infinium Human Methylation EPIC BeadChip array (850K) v1.0 (hereafter referred as EPIC) in line with the producer’s protocol (Illumina, San Diego, CA, USA) at the Max Planck Institute in 2020.

QC and normalization of the EPIC DNAm raw data were conducted using the meffil pipeline R package [37] (https://github.com/perishky/meffil/wiki). From the available blood DNA samples, three were excluded (3/146) and from adipose tissue DNA samples, three were excluded (3/113) as sex-mismatch or outliers based on the QC report. Furthermore, CpG site outliers were limited with winsorizing technique (1% each side; 2 values per CpG each side). Cell composition in blood (Bcell, CD, CD8T, Eos, Mono, Neu, and NK) was estimated with the meffil package [37]. Cell composition in adipose tissue was estimated with a reference free method available in RefFreeEWAS R package [38,39] (https://rpubs.com/paujedynak/reffree_cell_mix_tutorial) producing four cell types in our data.

### 2.4. Covariates

Covariates were chosen based on the previous literature [13,40] and relevant covariation between sibling pairs’ phenotype or DNAm patterns. Data on maternal smoking during pregnancy was collected from hospital records. Participants filled in questionnaires concerning personal health, e.g., smoking status. The DNAm batch effect (sample plate) was adjusted for in the analyses.

### 2.5. Statistics

Descriptives of the sibling pairs were examined according to distributions with paired samples t-test or McNemar Change test. Normal distribution was visually inspected by QQ-plot. DNAm data was applied as beta values. We applied mixed effects model and paired sample t-test adjusted for age, sex, batch, estimated cell composition, maternal smoking, BMI, and smoking to investigate DNAm differences between participants and sibling controls. We corrected the results for multiple testing by applying the false discovery rate (FDR) <0.05 with Benjamini and Hochberg procedure [41] as well as applying an epigenome-wide significance threshold of <9 × 10^−8^ [42]. QQ-plot of the blood EWAS showed some deflation, which was consistent with a low lambda value (0.85) (Supplemental figure S1). The adipose tissue EWAS showed no genomic inflation (Supplemental figure S2). Sensitivity analyses were performed as such that only matched sibling pairs were used and additionally by excluding preterm/SGA controls from the analyses. A follow-up analysis of 66 sites observed originally in the HeSVA study [43] and 12 sites observed in the NZ VLBW study [16] was conducted, and significant replication was defined at FDR < 0.05. Of the 66 sites found in the HeSVA study, 4 were not available in the Sibling study’s EPIC data (cg15374751, cg09854317, cg01886524, and cg13326175).

To gain insight into the biological processes associated with the observed differential DNAm, we conducted Ingenuity Pathway Analysis (IPA, QIAGEN, 2025, Version 150276282) [44]. We applied 458 significant sites from adipose tissue with annotated genes. This resulted in 393 CpG sites with known gene locations. Where multiple CpG sites were annotated to one gene, we selected the site with the lowest *p*-value, ultimately identifying 293 unique genes to use as input for IPA. Since IPA was originally developed for gene expression data, methylation coefficients were inverted, and thus hypermethylation was interpreted as downregulated and hypomethylation as upregulated gene expression. IPA applied Fisher’s Exact Test (FDR *p* < 0.05) to identify significant pathways.

We used the IBM SPSS program for Windows, version 29.0.1.1 (244) (IBM, Chicago, IL, USA), R (4.5.0), and RStudio software (2025.05.0.1 Build 513) [45] in CSC ePouta platform (research.csc.fi).

## 3. Results

Characteristics of the study population are shown in Table 1. When the birth data of the sibling controls was verified from birth records, four turned out to have been born preterm and two as SGA but all controls had a birth weight above 2100 g, and all were included in the analyses. Other maternal and participant characteristics were similar between VLBW participants and controls, except for impaired glucose regulation [31] and asthma which were more prominent in VLBW than sibling controls (Table 1). Medication reported by the participants is specified in Supplemental Table 1.

**Table 1.** Participant characteristics of VLBW individuals in comparison to their sibling controls.

	VLBW (*n* = 75)	Sibling controls (*n* = 73)	
	Mean (SD)		*P* value
Maternal age, years	29.7 (5.0)	29.8 (5.1)	0.81
Maternal smoking, yes, *n*/total *N* (%)	11/74 (14.9)	12/67 (17.9)	1.00
Gestational age, wk	29.6 (2.4)	39.6 (1.5)	< 0.001
Preterm, *n*/total *N* (%)^1^	75/75 (100)	4/73 (5.5)	< 0.001
Birth year, range	1978–1990	1973–1996	
Sex, female, *n*/total *N* (%)	39/75 (52.0)	38/73 (52.1)	0.99
Birth weight, g	1156 (215)	3372 (452)	< 0.001
Birth weight, SDS	−1.34 (1.61)	−0.35 (0.91)	< 0.001
SGA, *n*/total *N* (%)	27/75 (36.0)	2/73 (2.7)^2^	< 0.001
Adult age, years	29.4 (2.6)	29.4 (5.0)	0.84
Adult BMI, kg/m^2^	24.4 (4.8)	24.9 (4.5)	0.36
Adult smoking, yes, *n*/total *N* (%)	20/74 (27.0)	23/73 (31.5)	0.52
Hazardous alcohol consumption^3^, yes, *n*/total *N* (%)	20/74 (27.0)	26/72 (36.1)	0.07
Diabetes^4^, yes, *n*/total *N* (%)	2/75 (2.7)	1/71 (1.4)	0.25
Impaired glucose regulation^5^, yes, *n*/total *N* (%)	22/69 (31.9)	11/67 (16.4)	0.011
Hypertension^6^, yes, *n*/total *N* (%)	7/70 (10.0)	6/68 (8.8)	0.10
Asthma^7^, yes, *n*/total *N* (%)	14/70 (20.0)	7/66 (10.6)	< 0.001
ADHD^7^, yes, *n*/total *N* (%)	2/69 (2.9)	0/66 (0)	0.50

Differences between groups tested with paired samples T-test or McNemar Change test.

^1^Preterm: born at <37 gestational weeks; four preterm sibling controls who were born between gestational weeks 35.9 and 36.9.

^2^2 SGA sibling controls’ birth weight above −2.6 SDS.

^3^Based on self-reported questionnaire’s total Audit scores. Category includes hazardous or harmful alcohol consumption and moderate to severe alcohol use disorder.

^4^Type 1 or type 2 diabetes based on self-reported questionnaire or OGTT, all three identified are type 2 diabetes.

^5^Includes diabetes, impaired glucose tolerance and impaired fasting glucose based on OGTT.

^6^Based on self-reported questionnaire or three blood pressure measurement with a threshold of 140/90 mmHg.

^7^Based on self-reported questionnaire.

VLBW, born preterm with very low birth weight (<1500 g); SDS, standard deviation score; SGA, small for gestational age, defined as birth weight below −2.0 SDS based on national normative data.

Abbreviations: ADHD, attention deficit hyperactivity disorder; OGTT, oral glucose tolerance test.

### 3.1. Blood DNAm

**Figure 2.**
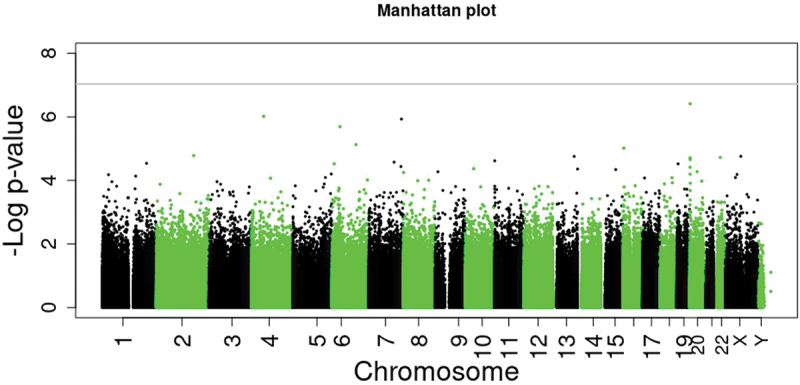
Manhattan plot of epigenome-wide association study (EWAS) with 862 475 CpG sites in blood comparing adults born preterm with very low birth weight (VLBW) and their sibling controls. Grey line presents *p* value < 9 ×10^−8^ adjusted for sex, age, batch, estimated cell composition, maternal smoking, BMI, and smoking. No CpG sites presented differential methylation with *p* < 9 ×10^−8^ nor FDR-corrected *p* < 0.05.

None of the 862 475 CpG sites studied in blood differed in methylation between VLBW and their sibling controls, applying a *p* value threshold of FDR <0.05 or a threshold of *p* < 9 × 10^−8^, adjusted for sex, age, batch, estimated cell composition, maternal smoking, BMI, and smoking (Figure 2 and Table 2). Table 2 shows the top 10 DNAm-sites between VLBW and sibling controls. Sensitivity analyses with limiting individuals to matched sibling pairs (*n* = 132; 66 pairs), and excluding six preterm and SGA controls (n = 137) both confirmed no significant associations.

**Table 2.** Top 10 sites for blood DNA methylation difference in VLBW (*n* = 71) compared with sibling controls (*n* = 72).

CpG	Chr	Gene	Coefficient (SE)	*p* value	FDR-adjusted *p* value
cg05857996	20	*EBF4*	0.131 (0.025)	3.88E^−07^	0.33
cg06035798	4	NA	0.026 (0.005)	9.59E^−07^	0.41
cg19080839	7	*SMARCD3*	0.031 (0.006)	1.17E^−06^	0.34
cg25325512	6	*PIM1*	−0.048 (0.010)	2.03E^−06^	0.44
cg00587628	6	*MFSD4B*	−0.007 (0.002)	7.48E^−06^	1.00
cg05501756	16	*SRL*	0.026 (0.006)	9.64E^−06^	1.00
cg17064434	2	NA	0.026 (0.006)	1.65E^−05^	1.00
cg05152459	X	*MAGEE2*	−0.035 (0.008)	1.75E^−05^	1.00
cg02867869	13	*SLC15A1*	0.056 (0.013)	1.75E^−05^	1.00
cg16686960	22	*TCN2*	0.016 (0.004)	1.88E^−05^	1.00

Coefficient presents methylation change in VLBW participants compared with controls. Mixed effect model applied adjusting for age, sex, batch, estimated cell composition, maternal smoking, BMI, and smoking.

VLBW, born preterm with very low birth weight.

### 3.2. Adipose tissue DNAm

**Figure 3.**
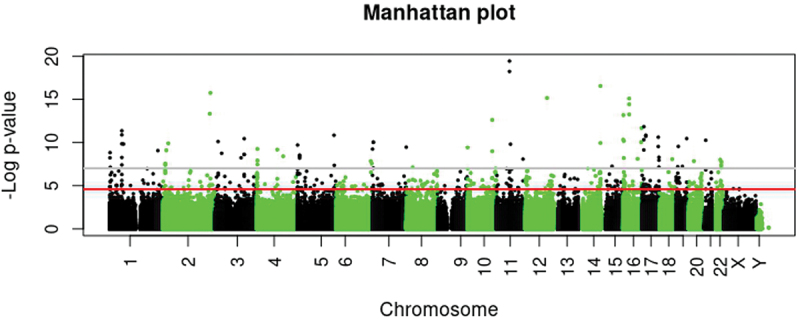
Manhattan plot of epigenome-wide association study (EWAS) with 862 698 CpG sites in adipose tissue comparing adults born preterm with very low birth weight (VLBW) and their sibling controls. Grey line presents *p* value <9 ×10^−8^ with 86 differential CpG sites, and red line shows FDR-corrected *p* < 0.05 with 458 differential CpG sites adjusted for sex, age, batch, estimated cell composition, maternal smoking, BMI, and smoking.

In adipose tissue, 458 out of 862 698 CpG sites differed between VLBW and their sibling controls (FDR *p* < 0.05), and 84 CpG sites with a threshold of *p* < 9 × 10^−8^ (Figure 3, Table 3 and Supplementary Data 1). Of the 458 sites, 432 (94%) were hypermethylated in VLBW compared with sibling controls (Table 3). In sensitivity analyses with limiting individuals to matched sibling pairs (*n* = 106; 53 pairs) showed a decline in the number of differential sites between cases and controls; 351 CpG sites differed with FDR adjustment and 67 sites with epigenome-wide significance (Supplementary Data 2). We observed only small changes when excluding two SGA controls (*n* = 108) from analyses; 451 CpG sites differed with FDR adjustment and 87 sites with epigenome-wide significance, with no relevant change in the top sites. Supplementary Data 1 shows 261/458 persisting sites after these sensitivity analyses. None of the significant sites observed in adipose tissue were associated with blood DNAm.

**Table 3.** Top 30 sites for fat DNA methylation difference in VLBW (*n* = 55) compared with sibling controls (*n* = 55).

CpG	Chr	Gene	Coefficient (SE)	*p* value	FDR-adjusted *p* value
cg00264176	11	*FADS2*	0.077 (0.007)	3,76E^−20^	3,24E^−14^
cg07999042	11	*FADS2*	0.072 (0.006)	5,97E^−19^	2,57E^−13^
cg08277679	14	*KIF26A*	0.053 (0.005)	2,86E^−17^	8,22E^−12^
cg14157824	2	*ACSL3*	0.050 (0.005)	1,79E^−16^	3,86E^−11^
cg25227378	12	NA	0.038 (0.004)	6,93E^−16^	1,20E^−10^
cg05127221	16	*QPRT*	0.063 (0.007)	8,05E^−16^	1,16E^−10^
cg03488456	16	*QPRT*	0.053 (0.006)	3,74E^−15^	4,61E^−10^
cg13394859	2	*LINC02832*	0.034 (0.004)	4,68E^−14^	5,04E^−09^
cg01468711	16	*QPRT*	0.048 (0.006)	5,67E^−14^	5,43E^−09^
cg04014417	16	*SRRM2*	0.050 (0.006)	6,71E^−14^	5,79E^−09^
cg04325594	10	*AFAP1L2*	0.062 (0.007)	2,42E^−13^	1,90E^−08^
cg01242052	17	*PIK3R6*	0.046 (0.006)	1,46E^−12^	1,05E^−07^
cg25595867	16	*BANP*	0.050 (0.006)	2,13E^−12^	1,41E^−07^
cg01491990	1	*PLPP3*	0.048 (0.006)	4,34E^−12^	2,68E^−07^
cg10500503	1	*PLPP3*	0.027 (0.004)	1,31E^−11^	7,54E^−07^
cg10529470	5	*SH3PXD2B*	0.038 (0.005)	1,44E^−11^	7,76E^−07^
cg13721589	17	*SREBF1*	0.054 (0.007)	1,49E^−11^	7,56E^−07^
cg03340410	17	*SREBF1*	0.041 (0.005)	1,54E^−11^	7,38E^−07^
cg14127237	17	*SREBF1*	0.032 (0.004)	1,89E^−11^	8,59E^−07^
cg00017271	17	*BIRC5*	0.039 (0.005)	2,42E^−11^	1,05E^−06^
cg21046286	19	*NUP62*	0.031 (0.004)	3,48E^−11^	1,43E^−06^
cg12615535	3	*RBP1*	0.047 (0.006)	3,56E^−11^	1,40E^−06^
cg02205013	16	*MAPK8IP3*	0.035 (0.005)	4,69E^−11^	1,76E^−06^
cg21871213	17	*MYH10*	0.020 (0.003)	4,74E^−11^	1,70E^−06^
cg15729697	21	*MIR99A; MIR99AHG*	0.029 (0.004)	5,52E^−11^	1,91E^−06^
cg10258953	16	*MGRN1*	0.044 (0.006)	6,69E^−11^	2,22E^−06^
cg02161940	3	*RFTN1*	0.053 (0.007)	7,78E^−11^	2,49E^−06^
cg26593380	7	*FOXK1*	0.067 (0.009)	8,91E^−11^	2,75E^−06^
cg14289895	16	*AC092723.5*	0.044 (0.006)	9,07E^−11^	2,70E^−06^
cg07505373	7	*FOXK1*	0.047 (0.006)	9,59E^−11^	2,76E^−06^

Coefficient presents methylation change in VLBW cases compared with controls. Mixed effect model applied adjusting for age, sex, batch, estimated cell composition, and maternal smoking.

VLBW, born preterm with very low birth weight.

### 3.3. Follow-up analysis

We conducted follow-up analyses on the basis of previously published results from the HeSVA study [43] and the New Zealand 1986 very low birth weight cohort (NZ VLBW) [16], which compared blood DNAm in adults at VLBW and unrelated controls. Of HeSVA study’s 66 differential CpG sites, 62 were available in the Sibling study’s EPIC data. Of those 62 sites, 18 reached epigenome-wide significance in same direction in blood DNAm (32%) and 1 site in opposite direction in adipose tissue DNAm (1.6%) in the current study (Table 4). From the 12 differential sites observed in the NZ VLBW cohort, 8 sites were replicated in blood DNAm (67%) and none in adipose tissue DNAm in the Sibling study (Table 4). Out of these eight sites, seven were also observed in the HeSVA study and were located in genes: *HIF3A (Hypoxia Inducible Factor 3 Subunit Alpha)* (three sites: cg27146050, g22891070, cg16672562), *EBF4* (four sites: cg05825244, cg05857996, cg24263062, cg13518079) *and GLI2 (glioma-associated oncogene Family Zinc Finger 2)* (one site: cg20219891). CpG sites in *HIF3A* were hypomethylated and sites in *EBF4* and *GLI2* were hypermethylated in VLBW compared with controls. In addition, we tested whether the four differential sites found in neonatal blood in meta-analysis comparing low birth weight with normal birth weight [14] were present in the Sibling study. Three of these sites were available in our data (cg12929983, cg04844207, and cg25325512), and site cg25325512, annotated to gene *PIM1*, was replicated in the current study in blood (Table 2; FDR-adjusted *p*-value in follow-up analysis 6.11 × 10^−06^) but not in adipose tissue.

**Table 4.** The association results of a follow-up analysis of 62 CpG sites originally identified in the HeSVA study with shown 19 CpG sites replicated in the sibling study. In the HeSVA study, the sites were differentially methylated between VLBW and unrelated-controls and in the sibling study between VLBW and sibling-controls. DNAm was measured from blood (18/62 sites) and adipose tissue (1/62 sites).

			HeSVA study, blood samples		Sibling study, blood samples		
CpG site	Chr	Gene	Coefficient (SE)	*p*-value	Coefficient (SE)	*p*-value	FDR-adjusted *p*-value
cg16672562	19	*HIF3A*	−0.103 (0.015)	6.58E^−11^	−0.054 (0.020)	9.35E^−03^	0.032*
cg00637745	2	*LOC84931, GLI2*	0.092 (0.014)	3.15E^−10^	0.084 (0.025)	9.61E^−04^	0.006
cg27146050	19	*HIF3A*	−0.034 (0.005)	3.78E^−10^	−0.019 (0.007)	8.48E^−03^	0.032*
cg13872898	2	*LOC84931, GLI2*	0.057 (0.009)	8.18E^−10^	0.048 (0.018)	8.98E-^03^	0.032
cg20219891	2	*LOC84931, GLI2*	0.082 (0.013)	9.06E^−10^	0.067 (0.022)	3.60E^−03^	0.015*
cg17870997	2	*LOC84931, GLI2*	0.070 (0.011)	2.03E^−09^	0.064 (0.019)	1.02E^−03^	0.006
cg25325512	6	*PIM1*	−0.030 (0.005)	2.85E^−08^	−0.048 (0.010)	2.04E^−06^	0.00006
cg24263062	20	*EBF4*	0.062 (0.011)	3.24E^−08^	0.070 (0.018)	2.08E^−04^	0.002*
cg13518079	20	*EBF4*	0.069 (0.012)	3.59E^−08^	0.100 (0.023)	3.83E^−05^	0.0005*
cg14311362	2	*LOC84931, GLI2*	0.067 (0.012)	7.79E^−08^	0.053 (0.016)	1.24E^−03^	0.006
cg14959908	20	*EBF4*	0.029 (0.005)	9.06E^−08^	0.033 (0.008)	6.36E^−05^	0.0007
cg05857996	20	*EBF4*	0.055 (0.010)	1.93E^−07^	0.131 (0.025)	3.88E^−07^	0.00002*
cg16071681	18	*GATA6, CTAGE1*	0.024 (0.005)	1.11E^−06^	0.024 (0.007)	4.09E^−04^	0.003
cg05825244	20	*EBF4*	0.095 (0.019)	1.32E^−06^	0.104 (0.024)	2.20E^−05^	0.0004*
cg10784813	16	*SOCS1*	0.018 (0.004)	1.49E^−06^	0.020 (0.006)	1.71E^−03^	0.008
cg09718582	2	*LOC150935, MIR4786*	0.024 (0.005)	1.87E^−06^	0.029 (0.008)	2.99E^−04^	0.002
cg22171829	7	*PDK4*	−0.028 (0.006)	4.36E^−06^	−0.031 (0.009)	1.08E^−03^	0.006
cg06260709	20	*SNAI1, LINC00651*	0.026 (0.006)	4.50E^−06^	0.030 (0.010)	4.61E^−03^	0.019
					Sibling study, adipose tissue samples		
cg11024682	17	*SREBF1*	−0.012 (0.003)	5.79E^−06^	0.023 (0.005)	7.32E^−06^	0.0005

HeSVA (Helsinki Study of Very Low Birth Weight Adults) study’s [43] coefficient is changed from original publication to be correspondingly compared with the Sibling study (Adults Born Preterm Sibling). Coefficient indicate a change for VLBW compared with controls.

*Differentially methylated CpG site in NZ VLBW (New Zealand 1986 very low birth weight) [16] cohort and replicated (8/12; 7 common shown) in the Sibling study.

VLBW, born preterm with very low birth weight.

### 3.4. Pathway analysis

**Figure 4.**
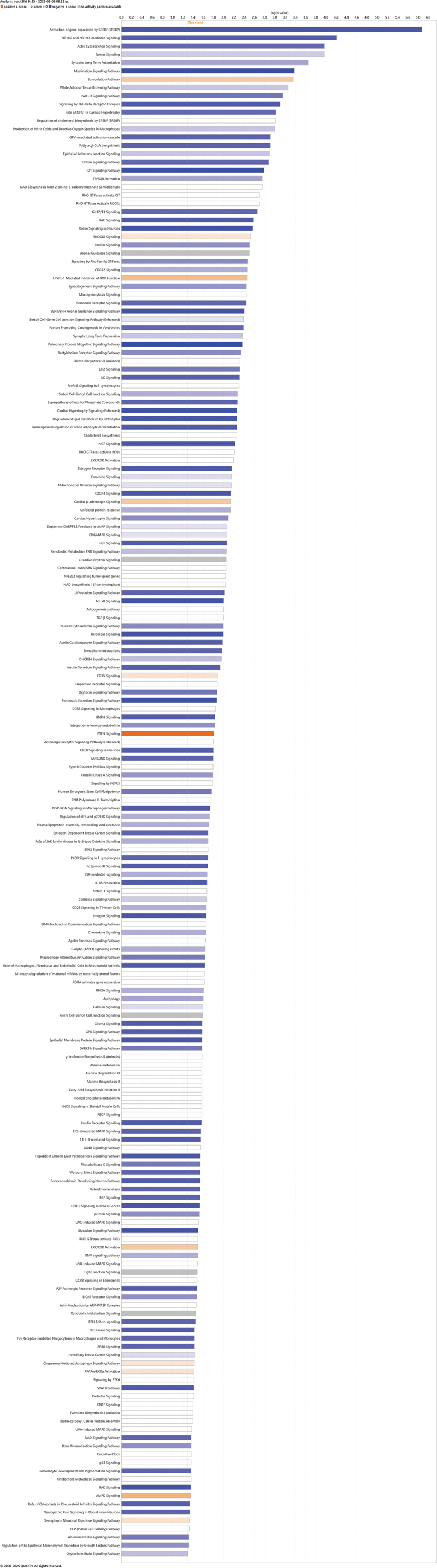
List of biological pathways identified via Ingenuity pathway analysis (IPA) based on differential CpG sites in adipose tissue. IPA identified 81 pathways suggesting possible mechanisms of VLBW birth leading to health outcomes. Orange line presents FDR-corrected p<0.05. Blue colors refer negative z-score and pathway inhibition, the darker blue the more inhibited; Orange colors refer positive z-scores and pathway activation, the darker orange the more activation; White color refers to z-score at 0 or close to 0; Grey color refers to no prediction possible. SREBP (SREBF), Sterol Regulatory Element Binding Transcription Protein/Factor; NR1H2/3, Nuclear Receptor Subfamily 1 Group H Member 2/; NAFLD, Non-Alcoholic Fatty Liver Disease; TGF, Transforming Growth Factor; NFAT, Nuclear Factor of Activated T-cells, GPVI, Glycoprotein VI; ID1, Inhibitor of DNA Binding 1; TR/ RXR, Thyroid Hormone Receptor/Retinoid X Receptor; NAD, nicotinamide adenine dinucleotide; CIT, Citron kinase; ROCKs, Rho-associated coiled-coil containing protein kinases; Gα12/13, heterotrimeric G protein subunits; RAC, GTP-binding protein; RHOGDI, RHO GDP Dissociation Inhibitor; CDC42, Cell Division Cycle 42; LPS, Lipopolysaccharide; IL-1, Interleukin-1; RXR, Retinoid X Receptor; WNT/SHH, Wingless/Sonic Hedgehog; EIF2, Eukaryotic Initiation Factor 2; ILK, Integrin-Linked Kinase; PPARalpha, Peroxisome Proliferator-Activated Receptor Alpha; NGF, Nerve Growth Factor; PKNs, Protein Kinase Ns; LXR/RXR, Liver X Receptor/Retinoid X Receptor; CXCR4, Chemokine receptor 4; DARPP-32, Dopamine- and cAMP-Regulated Phosphoprotein of 32 kDa; ERK/MAPK: Extracellular signal-Regulated Kinase/Mitogen-Activated Protein Kinase; HGF, Hepatocyte Growth Factor; PXR, Pregnane X Receptor; NFE2L2, Nuclear Factor Erythroid-Derived 2-Like 2; NF-κB, Nuclear Factor kappa-light-chainenhancer of activated B cells; TGF-β, Transforming Growth Factor-beta; DHCR24, 24-dehydrocholesterol reductase; CDK5, Cyclin-dependent kinase 5.

**Figure 5.**
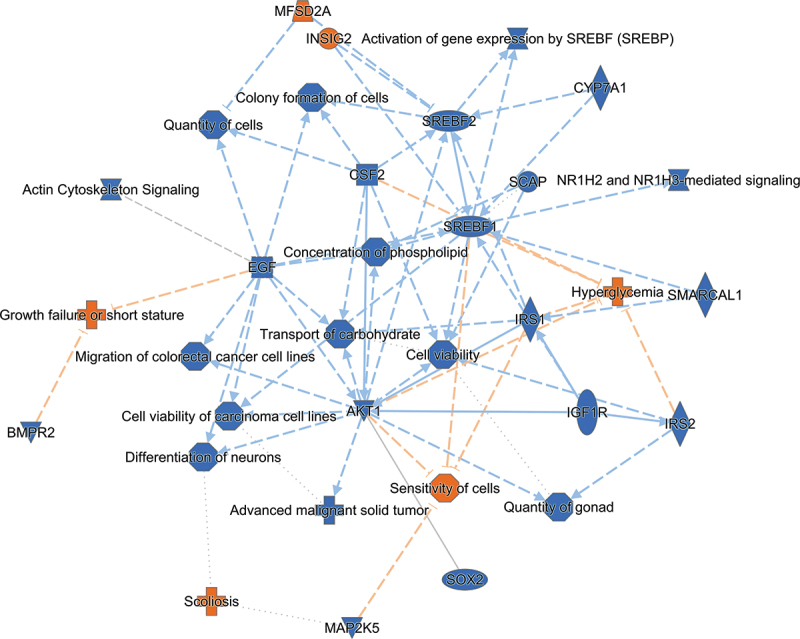
Summary graph of possible biological pathways identified via Ingenuity pathway analysis (IPA) based on differential CpG sites in adipose tissue of VLBW adults in comparison with their sibling controls. Blue color refers to pathway inhibition and blue line indicates leading to inhibition; Orange color refers to pathway activation and orange line indicates leading to activation. CYP7A1, cytochrome P450 family 7 subfamily A member 1; INSIG2, insulin induced gene 2; ACACB, acetyl-CoA carboxylase beta; SREBF1/2, sterol regulatory element binding transcription factor 1/2; SCAP, SREBF chaperone; NR1H2/3, Nuclear Receptor Subfamily 1 Group H Member 2/3; MFSD2A, MFSD2 lysolipid transporter A, lysophospholipid; SMARCAL1, SWI/SNF related, matrix associated, actin dependent regulator of chromatin, subfamily a like 1; BMPR2, bone morphogenetic protein receptor type 2; IRS1/2, insulin receptor substrate 1/2; AKT1, AKT serine/threonine kinase 1; CSF2, colony stimulating factor 2; EGF, epidermal growth factor; IGF1R, insulinlike growth factor I receptor; SOX2, SRY (sex determining region Y)-box 2; MAP2K5, mitogen-activated protein kinase 5.

We conducted an IPA with differentially methylated sites with a known gene location from adipose tissue. Hypermethylation at the CpG site was interpreted as downregulated gene expression. IPA identified 81 pathways (Fisher’s Exact Test, FDR *p* < 0.05) with a majority (58/81), showing possibly downregulation (Figure 4). The top five canonical pathways identified to be downregulated in adults at VLBW compared with sibling controls were activation of gene expression by *SREBP (SREBF) (Sterol Regulatory Element Binding Transcription Protein/Factor), NR1H2* and *NR1H3 (Nuclear Receptor Subfamily 1 Group H Member 2 and 3)*-mediated signaling, Actin Cytoskeleton Signaling, Netrin Signaling, and Synaptic Long-Term Potentiation (Figure 4). Figure 5 summarizes possible cascades between VLBW birth and health outcomes suggested by IPA based on differential methylation in the adipose tissue.

## 4. Discussion

We conducted a sibling control study to examine the association between extreme exposure of preterm birth at VLBW and epigenome-wide DNAm in blood and adipose tissue approximately 30 years after birth. In adipose tissue, we discovered 458 differentially methylated CpG sites between adult VLBW compared with their sibling controls. No DNAm differences were observed in blood as such, but our study replicated seven previously discovered CpG sites located in *EBF4, HIF3A,* and *GLI2*. Most of the sites were hypermethylated in VLBW. Top CpG sites in adipose tissue showed hypermethylation in gene locations, including *FADS2, KIF26A, PLPP3, QPRT*, and *SREBF1*, suggesting possible inhibition of lipid metabolism, neurological function, and cell cycle control. Pathway analysis identified predicted inhibition in lipid synthesis and metabolism as well as neurological regulation and cardiac development and metabolic regulation pathways in VLBW individuals compared with siblings. The findings were observed in adipose tissue and imply possible mechanisms between VLBW and later poor cardiometabolic and neurological health outcomes.

Previous studies on early life exposures and DNAm have mainly focused on blood samples, which are more readily accessible and less invasive to collect than other tissue samples. These observations indicate that the extensively differential DNAm at neonatal age in relation with birth weight or gestational age may be partly resolved by adulthood [13,14,16,46,47]. However, few changes seem to persist or are present in adulthood. Our study identified fewer differentially methylated sites in blood as compared to our previous findings from HeSVA study [43]. This might suggest the presence of residual confounding (e.g., familial environment or genetic confounding), when comparing cases with unrelated controls [48].

The follow-up analysis, consisting of investigating previously discovered sites in blood, replicated in seven sites located at *EBF4, HIF3A,* and *GLI2*. *EBF* transcriptional family is involved in neural development and B-cell maturation [49], and *EBF4* might regulate immune functions [50]. Further, hypermethylation in *EBF4* is associated with BMI in adults [51] and possibly with higher mortality [50]. *HIF3A* regulates responses to hypoxia and probably in lung function and development [52], as well as adipose tissue differentiation [53] and dysfunction [54]. Hypermethylation of *HIF3A* in cord blood is associated with higher gestational age [55] and higher blood pressure in childhood [56]. Furthermore, higher DNAm in *HIF3A* is associated with obesity in children [57] and BMI in adult blood and adipose tissue [58,59], which suggest a role in metabolic processes. Another gene of interest is *GLI2*, which is involved in embryonic development, multiple organ development, and cell differentiation and may play a role in cardiovascular diseases by downregulated activity possibly leading to less vascular calcification [60,61]. In relation to this, *GLI2* mediates Sonic Hedgehog signaling, neurological disorders [62], and bone formation [61].

Of the seven replicated sites in blood from the HeSVA and NZ VLBW cohorts, four were associated with gestational age in the meta-analysis [13]; cg27146050 *(HIF3A*, hypomethylated), cg16672562 *(HIF3A*, hypermethylated), cg13518079 *(GLI2*, hypermethylated), and cg20219891 *(GLI2*, hypermethylated). However, not all associations were in the same direction as in the current study. Higher birth weight was associated with hypomethylation of cg20219891 *(GLI2)* in childhood in a meta-analysis [14]. Furthermore, methylation and reduced expression of *GLI2* in fetal brain have been associated with neural tube defect of the spina bifida [63]. In addition, site cg25325512, annotated to gene *PIM1*, was found to be differential in meta-analysis comparing low birth weight with normal birth weight [14], replicated in the current study in the same direction as in most cohorts included in the meta-analysis (hypomethylated). Above mentioned sites were not matched to sites observed in a small study of 12 participants born at extreme preterm birth in comparison with unrelated controls at birth nor as adults [46] or in differential sites observed in buccal cells in adults of extremely low birth weight [64]. In our study, *GLI2* located sites were hypermethylated and thus possibly downregulated. These findings may indicate possible mechanisms of VLBW and later health, as VLBW or very preterm adults display higher odds for cardiovascular diseases and related risk factors [2,31,35,65,66], neurological and mental health impairments [67–72], and lower bone mineral density [34,73,74].

In this unique cohort, we were able to obtain abdominal adipose tissue samples and found 458 differentially methylated CpG sites between adult VLBW and their sibling controls, with the majority of sites being hypermethylated in the VLBW group. Only a few studies have used adipose tissue samples up to date, but animal models suggest that prenatal diet has an impact in the offspring’s methylome and gene expression in adipose tissue [75,76]. Human studies have shown an association between adipose DNAm but not blood DNAm and adiposity phenotypes [77] and adipose DNAm and diet in adult men varying by birth weight [78,79].

We know that DNAm is tissue-specific. However, there is a lack of studies focusing on target tissues because of challenges in sample collection [80–82], limiting possibilities to explore the tissue-specific DNAm patterns. Tissue-specific differences in DNAm according to gestational age or birth weight were observed between cord blood and cord tissue, as well as between cord blood and infant saliva [83,84]. In contrast, another study reported similarities in DNAm between cord blood, fetal brain, and lung tissues [13]. We did not find overlapping sites in blood and adipose tissue in the Sibling study, but one site in adipose tissue was replicated from previous studies on blood (cg11024682 (*SREBF1*)). The top two differentially methylated sites (cg00264176; cg07999042) in adipose tissue were hypermethylated in the VLBW group and are annotated to the *FADS2* gene, in addition to four other differential sites (cg10069985; cg25324164; cg11250194; cg02213369). This protein coding gene is involved in the synthesis of important unsaturated fatty acids like docosahexaenoate (DHA) from essential polyunsaturated fatty acids. DHA is crucial for processes like nervous system development and function, and low levels have been observed in VLBW newborns [85].

Some of our findings on differential DNAm in adipose tissue may possibly reflect underlying metabolic disturbances, such as suboptimal lipid availability or inflammation, having relevance in adipogenesis and insulin resistance development [86,87] or dyslipidemia [88]. We have previously reported impaired glucose regulation, lower unsaturation in abdominal adipose tissue, and more centralized fat distribution in VLBW individuals compared to their sibling controls [31–33]. If replicated in other settings and linked to phenotypic changes, our DNAm findings could potentially serve as early biomarkers of disease in at-risk populations and help in finding therapeutic or interventional targets [86]. An epigenetic mark in adipose tissue might be long-lasting [89] potentially influencing long-term metabolic health.

The pathways in our study identified via IPA, based on differential methylation in adipose tissue, are related to neurological regulation and development, cellular processes, lipid metabolism, cardiac function, and metabolic regulation. Genes overlap with diseases such as metabolic syndrome, liver cancer, and coronary artery disease. The strongest pathway was a reduced gene expression by *SREBP (SREBF)*, which relates to downregulation of cholesterol, fatty acids, triglyceride, and phospholipid biosynthesis with possible effects on lipid metabolism as well as to glucose homeostasis in VLBW increasing odds for dys-/hyperglycemia [90]. Furthermore, four sites in *SREBF1* and 1 in *SREBF2* were differentially methylated in adipose tissue, and one site was replicated from a previous VLBW study on blood. *SREBF1* may affect atherogenesis [91] and bone metabolism [92]. Methylation of *SREBF1*, although not in the matched CpG site of our study, affected BMI and was associated with glycemic traits, dyslipidemia, and coronary artery disease in a large set of adult cohorts [59]. *SREBF2* is also associated with downregulation of *PPARG (peroxisome proliferator activated receptor gamma)* [93], a key factor in adipocyte differentiation. Downregulation by hypermethylation of *PPARG* in mouse adipocytes and fat tissues indicated a pathway for metabolic syndrome [94] and may be altered in low birth weight human adults [79]. Targeted methylation of *PPARG* in subcutaneous fat tissue seems to be associated with higher body and visceral fat mass in adults [95]. *PPARG* also has a role in regulating fatty acid oxidation, glucose homeostasis, and may lead to short stature [96,97]. We and others have reported shorter height and lower lean body mass in individuals born at VLBW compared with controls [34,98,99]. In these genes and pathways, there may exist possibilities of reversing the detrimental pathway by dietary modification [100].

Fatty acyl-CoA biosynthesis is essential in fatty acid synthesis, and it is essential for various biological processes such as building blocks of cellular membranes, energy storage, and cell signaling [101,102]. Possible downregulation of fatty acyl-CoA biosynthesis pathways was observed in the VLBW participants compared with sibling controls. Possibly downregulated in VLBW adults, the *NR1H2* and *NR1H3 (Nuclear Receptor Subfamily 1 Group H Member 2 and 3)*-mediated signaling regulates cholesterol, bile acid, and triglyceride metabolism, gluconeogenesis and has implications in inflammation processes [103]. These findings may contribute to altered lipid profile, possibly associated with higher cardiovascular disease risk and neurodevelopment, which may occur in VLBW already at birth [85,104]. Indeed, dysfunction of the adipose tissue in VLBW may be one underlying mechanism of increased disease risk in later life [105].

Another pathway potentially inhibit in VLBW in the current study was the orexin signaling pathway, which is involved in many biological processes, including adipogenesis, but also in neurological functions, such as appetite control, sleep-wake regulation, mental health, and neuroinflammation [106]. Also, serotonin receptor signaling, which also has significance in mood, appetite, sleep-wake cycle, and cognition among others [107], was identified as downregulated in VLBW. We have previously observed an earlier chronotype in VLBW than sibling controls [30]. Reversing detrimental effects of VLBW by dietary intake may be possible, as suggested by a study of VLBW infants followed up until 5 years of age, where breastfeeding was associated with DNAm with key sites linked to neurodevelopmental outcomes [108].

This study has several strengths, including the use of a sibling control design, which strengthens our conclusions by minimizing confounding of genetic and early environmental exposures. Further, we have rare tissue samples of both blood and subcutaneous adipose tissue. Another strength of the current study design is that we are examining more stable adult samples in contrast to neonatal samples, in which observed DNAm differences might reflect cell-type changes due to fetus and infant developmental age [46]. There are also some limitations. The number of study participants was limited as expected when having longitudinal follow-up with extreme early childhood exposure of VLBW, which may compromise statistical power [109,110]. Blood EWAS showed a tendency toward deflation, possibly due to its small sample size. By design, the study was limited to VLBW participants with same-sex siblings, both volunteering for an extensive assessment, thus limiting the generalizability of the results. IPA included an assumption that hypermethylation reflects inhibition or downregulation of specific genes; however, this is not always the case, and thus the IPA results must be interpreted with caution. Uncertainties rely on the estimation of cell types in adipose tissue, and the possibility of unmeasured cell-type effect remains [111]. DNA sequence polymorphism can influence DNAm by 13–80% depending on factors such as cell type [80], which we were unable to account for in this study.

## 5. Conclusions

In conclusion, we found evidence to support differential DNA methylation in subcutaneous adipose tissue of adults born preterm at VLBW, compared with sibling controls. The differential DNAm may indicate long-term alterations in adipose tissue function programmed during early development. Such changes may partially account for the observed variations in metabolic traits and the increased risk of chronic diseases in adults born preterm.

## Supplementary Material

Supplemental Material

## Data Availability

Restrictions apply to the availability of individual-level data to preserve patient confidentiality. Upon reasonable request to the corresponding author, partial data may be provided. Metadata of the study can be found in Qvain repository (https://doi.org/10.23729/fd-8e6178a7-f368-3d38-a28b-e16c911a6408).

## Article highlights

- The Developmental Origins of Health and Disease (DOHaD) hypothesis suggests that early life exposures can cause lasting changes in organ structure and function, impacting, e.g., cardiometabolic health. One proposed mechanism for these changes is epigenetic modifications, specifically DNA methylation (DNAm). We compared epigenome-wide DNAm via Illumina EPIC 850K in blood and adipose tissue of adults born preterm with very low birth weight (VLBW) and their sibling controls.
- Results showed no DNAm differences in blood samples across the entire cohort; however, follow-up analyses revealed seven differential CpG, annotated to genes *EBF4, HIF3A*, and *GLI2*.
- In adipose tissue, we observed substantial differences in DNAm, with 458 CpG sites differentially methylated (FDR *p* < 0.05) between VLBW individuals and their siblings, the majority of which were hypermethylated in VLBW.
- Ingenuity pathway analysis identified 81 possible biological pathways related to fat metabolism, neurological regulation and development, cardiac function and development, and cellular regulation, suggesting possible mechanisms of preterm birth at VLBW and long-term health.
- The study findings underscore the potential role of DNAm in adipose tissue as a mediator of health risks associated with preterm birth and VLBW. These epigenetic findings may provide the potential to develop targeted interventions.
