# Supplementary material for: Blood and adipose tissue DNA methylation in adults born preterm with a very low birth weight – a sibling comparison study
Source: Epigenomics. 2025 Nov 17;18(1):1–14. doi: 10.1080/17501911.2025.2583893 (PMC12826731; doi:10.1080/17501911.2025.2583893)
Supplement: Supplemental Material [file IEPI_A_2583893_SM1158.zip › suppl_data/Supplementary table 1.docx]

| Supplementary table 1 Reported medication use in VLBW individuals and their sibling controls | | |
| --- | --- | --- |
|  | VLBW (n=75) | Sibling controls (n=73) |
| Indication or category of medication use | | |
| Allergy | 9 | 8 |
| Topical corticosteroids | 6 | 4 |
| ADHD | 0 | 1 |
| Psychiatric | 8 | 5 |
| Other | 21 | 10 |
| Average number (SD) of medications used | 1.8 (1.4) | 1.7 (1.3) |
| Data is based on self-reported questionnaire. None reported antihypertensive medication.  VLBW, born preterm with very low birth weight (<1500 g); ADHD, attention deficit hyperactivity disorder | | |
