# Supplementary figures and images for: Blood and adipose tissue DNA methylation in adults born preterm with a very low birth weight – a sibling comparison study
Source: Epigenomics. 2025 Nov 17;18(1):1–14. doi: 10.1080/17501911.2025.2583893 (PMC12826731; doi:10.1080/17501911.2025.2583893)

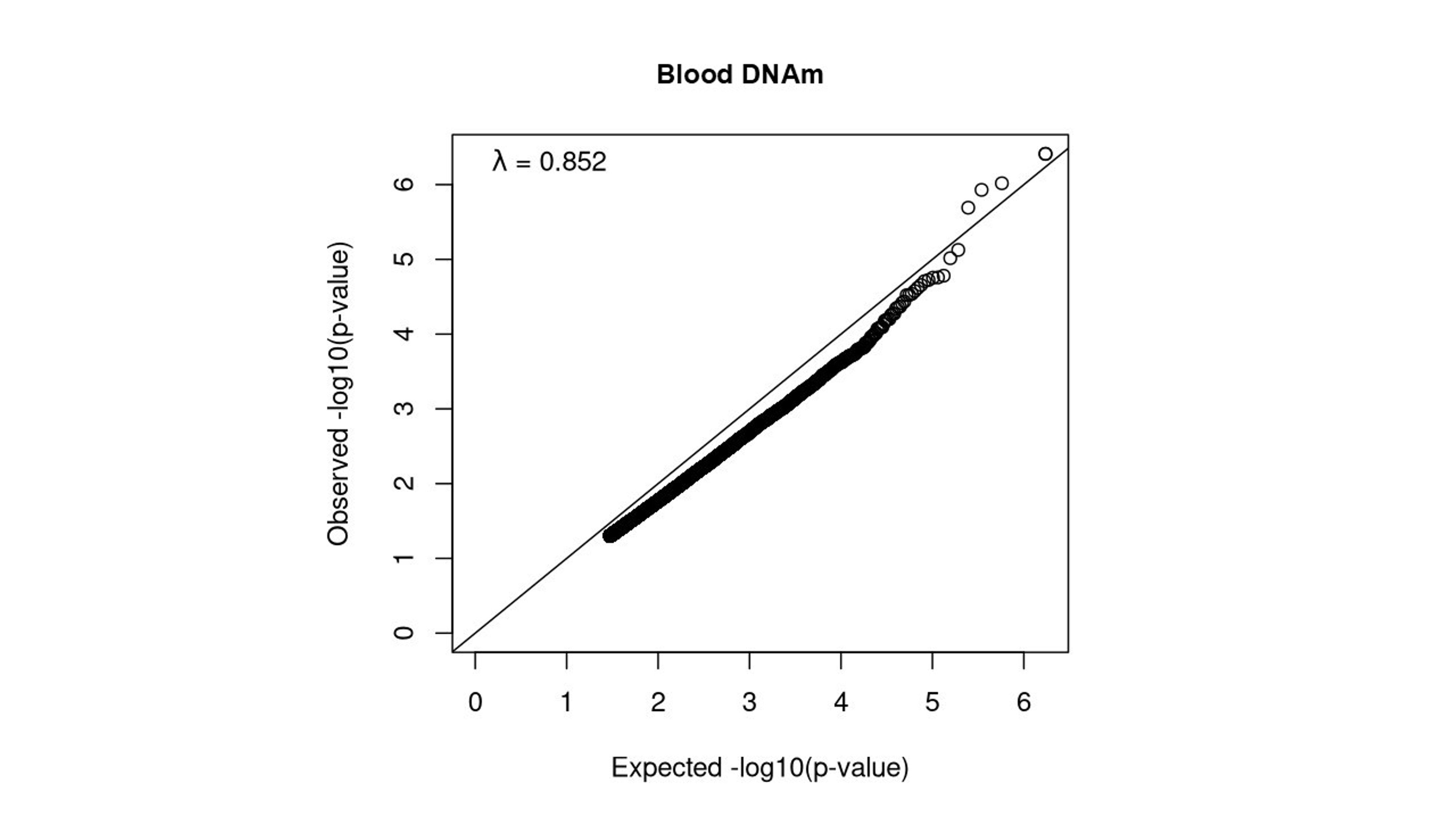

Supplement: Supplemental Material [file IEPI_A_2583893_SM1158.zip › suppl_data/Supplementary_Fig_1_qqplot_blood.png]

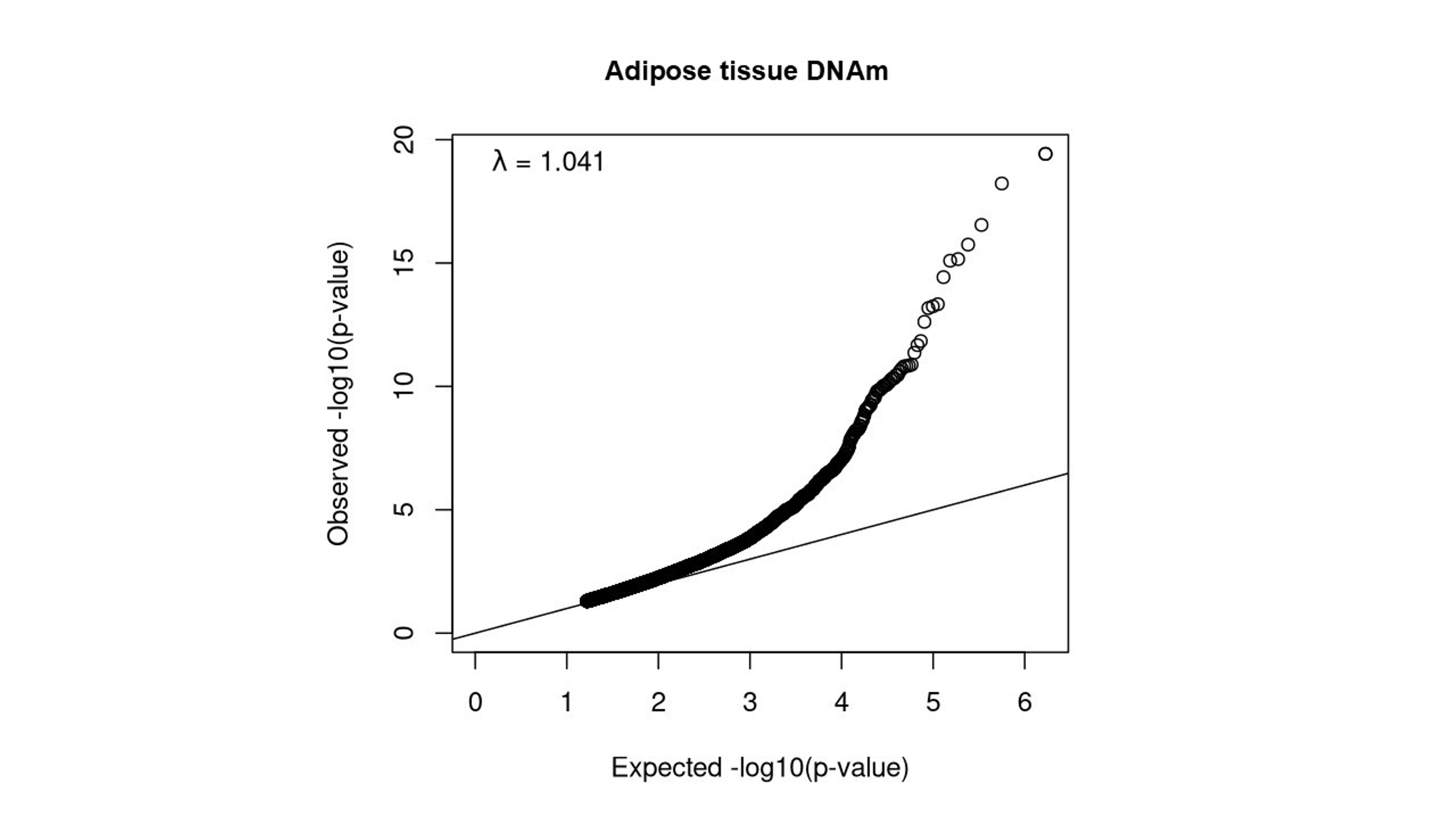

Supplement: Supplemental Material [file IEPI_A_2583893_SM1158.zip › suppl_data/Supplementary_Fig_2_qqplotadipose.png]
